# The Integrated Approach to Inherited Disorders in Neurotransmitters from Molecules to Systems

**DOI:** 10.3390/ijms232415974

**Published:** 2022-12-15

**Authors:** Mariarita Bertoldi, Patrizia Polverino de Laureto

**Affiliations:** 1 Department of Neuroscience, Biomedicine and Movement Sciences, University of Verona, Strada Le Grazie 8, 37134 Verona, Italy; 2 Department of Pharmaceutical and Pharmacological Sciences, University of Padova, Via Marzolo 5, 30131 Padova, Italy

This Special Issue focusses on monoamine neurotransmitters responsible for mediating neuronal transmission. They represent a heterogeneous class of compounds, including catecholamines (dopamine, adrenaline, and noradrenaline), indoleamines (serotonin and tryptamine), amino acids (γ-aminobutyric acid (GABA), glycine, aspartate, and glutamate), and acetylcholine. They are involved in disorders such as Parkinson’s and Alzheimer’s disease, depression, and other neuropsychiatric diseases in adults. In children, the great majority of developmental disorders such as epilepsy, autism, ADHD, learning and intellectual disabilities, and movement disorders are linked to alterations in the level of monoamine neurotransmitters. Mutations in the genes coding for enzymes/proteins related to neurotransmitter synthesis, metabolism, and transport lead to a variety of inherited deficiencies, globally referred to as neurometabolic disorders. Although some markers for the pathological states due to monoamine neurotransmitter lack or alteration are known, widespread efforts are needed to improve our understanding of pathophysiological mechanisms to design more appropriate systemic approaches. Some of these disorders can be treated pharmacologically; in other cases, the current therapies are only palliative, and thus the identification of specific monoamine pathway defects would enable the selection and development of disease-specific new drugs.

In this issue, the cross-sectional approach from molecules to mouse models has allowed us to look at neurotransmitter diseases from different points of view. Starting from an initial review of the impairment in the synthesis of dopamine and serotonin due to mutations in the *ddc* coding for aromatic amino acid decarboxylase (AADC) [1] that lead to severe pathological conditions of infantile parkinsonism, the main focus is on neurodegeneration and Parkinson’s disease (PD) as well as the regulation of epilepsy and neuroendocrine cells. Han [2,3] and Seo [4] studied the expression levels of various genes coding for tyrosine hydroxylase, serine/arginine-rich protein-specific kinase 3, and integrin α7 with respect to α-synuclein in SH-SY5Y and C2C12 cells as well as in a PD mouse model. Interestingly, all these proteins were less expressed in cells and the muscles of the mouse model, while α-synuclein expression was increased. These observations represent the basis of further investigations on other possible proteins that interplay in the manifestation of PD, not only in the regions of the brain affected by degeneration of the dopaminergic neurons but also at the periphery. These investigations can study muscle modifications and possibly relate them to movement disorders and impairment, a characteristic feature of PD.

Barak-Broker et al. [5] showed how phosphorylation regulates Ca^2+^ release in neuroendocrine cells through a biochemical experimental approach through dynamic FRET in PC12 cells and amperometric analyses of catecholamines release in the phospho-mimetic and phosphor-null of appropriate protein targets. This level of investigation conjugates biochemical analyses with other biological approaches that are evident in the article of Amakhin [6]. In this paper, a physiological approach based on whole-cell patch-clamp recordings in rat entorhinal cortex slices aimed to unravel the regulation of epileptic activity to Ca^2+^-permeable AMPA receptors, leading to the conclusion that the expression of these receptors in principal neurons could be considered a protective factor.

Overall, the complex world of neurotransmitter impairments could be tackled by differing expertise and point of view to build a more complete picture.

Three important lines of intervention can be individuated in future research on monoamine neurotransmitter disorders. 

A specific ambit of study concerns the relationship between the genotype and clinical phenotype. The knowledge of the correlation between missense mutations and altered protein function in different patients would provide insight into the pathology onset and progression, enabling the design of personalized medicine. 

A generation of new neuronal models could lead to increased knowledge at the molecular level of these brain disorders and provide platforms for high-throughput drug screening. 

The identification and development of new biomarkers for early diagnosis could improve the correct identification of the disease, often misdiagnosed, and therefore help develop specific and personalized therapy.
